# Zebrafish as an Emerging Model for Bioassay-Guided Natural Product Drug Discovery for Neurological Disorders

**DOI:** 10.3390/medicines6020061

**Published:** 2019-05-30

**Authors:** Arjun Pitchai, Rajesh Kannan Rajaretinam, Jennifer L. Freeman

**Affiliations:** 1 Molecular and Nanomedicine Research Unit (MNRU), Centre for Nanoscience and Nanotechnology (CNSNT), Sathyabama Institute of Science and Technology, Chennai 600119, Tamil Nadu, India; arjunbiotech@gmail.com; 2 School of Health Sciences, Purdue University, West Lafayette, IN 47907, USA

**Keywords:** Alzheimer’s disease, bioassay-guided purification, drug discovery, natural products, neurodegenerative disorder, neurodegenerative model, Parkinson’s disease, schizophrenia, transgenic, zebrafish

## Abstract

Most neurodegenerative diseases are currently incurable, with large social and economic impacts. Recently, there has been renewed interest in investigating natural products in the modern drug discovery paradigm as novel, bioactive small molecules. Moreover, the discovery of potential therapies for neurological disorders is challenging and involves developing optimized animal models for drug screening. In contemporary biomedicine, the growing need to develop experimental models to obtain a detailed understanding of malady conditions and to portray pioneering treatments has resulted in the application of zebrafish to close the gap between in vitro and in vivo assays. Zebrafish in pharmacogenetics and neuropharmacology are rapidly becoming a widely used organism. Brain function, dysfunction, genetic, and pharmacological modulation considerations are enhanced by both larval and adult zebrafish. Bioassay-guided identification of natural products using zebrafish presents as an attractive strategy for generating new lead compounds. Here, we see evidence that the zebrafish’s central nervous system is suitable for modeling human neurological disease and we review and evaluate natural product research using zebrafish as a vertebrate model platform to systematically identify bioactive natural products. Finally, we review recently developed zebrafish models of neurological disorders that have the potential to be applied in this field of research.

## 1. Introduction

Central nervous system (CNS) diseases and disorders, including Alzheimer’s disease (AD), schizophrenia (SCZ), Huntington’s disease (HD), and Parkinson’s disease (PD) [1,2,3], signify a global burden on society in terms of disability, economic loss, and human suffering. Globally, more than a million people have CNS disorders [4]. CNS disorders are multifaceted diseases with unclear causes and often ineffective therapies, with only a few therapeutic drugs being clinically effective [5]. Natural products (NPs) are small molecules synthesized from living organisms (plants, bacteria, and fungi) and are similar to secondary metabolites. Among all existing sources for drug discovery against single targets of new lead compounds [6], NPs are most promising but are underutilized. Crude extracts from NPs are a complex mixture of mostly uncharacterized compounds, some of which may have unwanted effects. Worldwide, nearly 30% of all top-selling drugs are NPs or their derivatives. NPs are an excellent source of new drug-like compounds to be discovered, and their diversity of chemicals has helped to develop drugs for a wide range of neurodegenerative disorders. Most new drugs have been authorized from either NPs themselves or NPs over the past 30 years [7,8,9]. In complex NP extracts, the isolation and structural characterization of bioactive small molecules involves several new methodologies that need considerable time and effort [10,11]. Furthermore, there are several methods involved in testing NPs in high-throughput screening (HTS). Combinatorial libraries with NP-like compounds have been recently used in HTS [9].

A vital component of the drug discovery program for NPs is bioassay-guided separation. In bioassay-guided separation, each chromatographic fractionation undertakes biological evaluation for further fractionation, and only biologically active fractions are selected. The crude extracts are fractionated and evaluated in bioassays. Further fractionation is repeated until the chosen activity isolates pure compounds and then characterizes them structurally. Novel pharmaceutically active NPs had been identified through screening and fractionation of crude extracts using several presently regarded in vitro assays, collectively with (i) cell fractions, (ii) entire cellular assays, or (iii) recombinant enzymes as target molecules [12]. Notwithstanding its application for HTS identification, the biomedical relevance of the isolated active metabolites can be limited when using only enzymatic or in vitro assays. To overcome this limitation, high-resolution micro-fractionation can be coupled with high-content bioassays to further analyze the separate constituents. In contrast to cell-based reporter or enzymatic assays, high-content bioassays (e.g., phenotypic assays using some cells or organisms) allow for an impartial investigation of pharmacological activity. Many in vivo animal models offer the possibility of independent screening of biomedically relevant bioactivities. However, milligrams of compounds are required for mammalian models and are thus not ideal for in vivo platforms for micro-fractionation and rapid HPLC profiling approaches.

Moreover, many naturally derived active compounds not only play a role as drugs but also help in the development of many new model structures for synthetic molecules through combinatorial chemistry. During the last 20 years, about 50% of drugs introduced to the market have been derived indirectly or directly from small bioactive molecules. As a source of chemical diversity, unfulfilled expectations from current R&D strategies and emerging trends have led to interest in NPs [7,13]. NPs have attracted considerable attention in the treatment of CNS diseases due to their neuroprotective and therapeutic effects. NPs are excellent sources of safe, precise, and effective anti-neurotherapeutic agents and thus are useful in the development of safer substitutes to pharmaceuticals. Recent literature suggests that many bioactive compounds have both neurotrophic and neuroprotective actions [14]; therefore, for peripheral neuropathy early treatment using phytochemical approaches could be one of the important strategies in preventing many neurological disorders.

Many presently known bioactive NPs have been previously recognized for their activity-guided extract isolation through the use of in vitro assays. Biologically active NPs have been identified by physical characteristics using chromatography, mass spectrometry, and NMR spectroscopy analysis. In vivo bioassay-guided fractionation has not widely been used for the discovery of drug-like NPs, as traditional in vivo models (e.g., mice and rats) are low-throughput systems and require much larger quantities of compounds for testing in these systems.

The zebrafish (*Danio rerio*) provides a complementary integrative biological model for the discovery of natural drug-like products through in vivo bioassay-guided chromatographic fractions requiring only microgram quantities of individual components. Zebrafish are vertebrates, and thus are more evolutionary similar to humans compared to non-vertebrate models. Logistically speaking, zebrafish are tiny and can be kept in a small space in high numbers. Zebrafish are currently emerging as an in vivo vertebrate model system for drug discovery and functional genomics [15]. In addition to their many pharmacological and physiological similarities with mammals, zebrafish have many added advantages including small size of embryos and larvae (0.5 to 5 mm depending on the stage of development), optical transparency, rapid ex vivo development, and high fecundity (up to hundreds of offspring per day). These characteristics makes zebrafish a standalone versatile experimental in vivo model compatible with HTS and NP discovery micro-fraction techniques [16]. Furthermore, zebrafish embryos and larvae provide the convenience of using microtiter plates (96-well and even 384-well plates) to test the activity of micro-fraction isolated natural compounds. Depending on the performance of these isolated compounds, the need for only microgram quantities to initiate an initial biological reaction represents another advantage of using zebrafish as a model organism in comparison to other vertebrates (e.g., rodents, where the energy dose requirements are typically one thousand times higher) [17]. This latter feature is prime to NP discovery, as many high-resolution HPLC-based separation techniques, particularly micro-fractionation, bring about very limited pattern quantities that could otherwise be inadequate for in vivo activity analysis.

The neuroprotective activity of bioactive compounds from herbal drugs has been proven by using cellular or animal models [18,19,20]. However, effective delivery of drugs to the brain remains the main task in the discovery and development of new CNS disease treatments [21,22]. This review focuses on neurological disorders with an emphasis on neurodegenerative diseases, use of zebrafish for bioassay-guided isolation of neuroactive small molecule from NPs, and new methods to develop zebrafish neurodegenerative models that have the potential for expansion into NP drug discovery applications.

## 2. Neurodegenerative Diseases

Neurodegenerative diseases lead to a rapid loss of brain processes such as cognitive and/or motor neuron function, and are a major challenge facing aging populations. AD, PD, HD, and amyotrophic lateral sclerosis (ALS) are common neurodegenerative diseases. Neurodegenerative diseases share common characteristics and mechanisms despite their different clinical forms. One of these features is regional cytosolic or nuclear protein aggregation [23]. Specific features include extra cell deposition of plaques of amyloid-beta (Aβ), intracellular accumulation of inclusions of hyperphosphorylated microtubule-binding tau in AD, intracellular storage of α-synuclein in PD, inclusion of TAR DNA-binding protein (TDP)-43 transactive response in ALS, frontotemporal dementia, and polyglutamine protein aggregates in HD and other repeat CAG-polyglutamine diseases. While for some cases genetic causes have been identified, the main driver is a complex interaction of predisposition factors in genetics and the environment. In every common neurodegenerative disease condition, there is usually a mixture of hereditary and "sporadic" forms. While the identity of many mutated genes in family forms of AD, PD, and ALS is known, the function of such genes and how their mutations induce neuronal degeneration is not fully understood. Processes that cause degeneration and the death of particular neuron types are probably the most important discovery goals in the field, shaping the disease’s manifestations and defining the characteristics of all neurodegenerative diseases.

## 3. Using the Zebrafish Model for Neurological Disorders

The zebrafish is being progressively used to model neurodegenerative diseases and neurological disorders successfully [24,25,26,27,28,29,30,31,32,33], with promises to test potential treatments for diseases and disorders [31,34]. The zebrafish CNS is similarly arranged to that of other vertebrates, and is traditionally separated into the hindbrain, midbrain, forebrain, ascending and descending spinal cord, cranial nerves, motor spinal cord, and sensory nerves. Zebrafish neuroanatomy has been examined and described in detail elsewhere during development, as well as in adults [35,36]. The genome of the zebrafish is widely annotated [37]. The evolutionary lineage of zebrafish (teleost-bonyfish) separated about 450 million years ago from the human lineage (tetrapod) [38]. Zebrafish pairs can produce large number of embryos that make it possible to achieve relatively high-throughput screening drug studies and behavioral testing [15] with simple methods for modulating gene expression available [39,40]. Many human-associated neurodegenerative disease proteins in zebrafish are homologous, highlighting potentially preserved molecular cellular functions that can be easily examined [28] (Table 1).

### 3.1. Zebrafish and Alzheimer’s Disease

The most common form of irreversible neurodegenerative disorder and dementia is Alzheimer’s disease (AD). Fifty million people were estimated to live with AD in 2018, and this figure is predicted to increase to 152 million by 2050 [62]. AD’s main clinical feature is progressive memory loss, motor and speech impairment, deception, depression, and aggressive behavior [63]. There is significant neuronal damage in AD patients in numerous brain regions [64,65]. This is usually caused by extracellular deposition of amyloid-beta peptide and tau protein aggregates called neurofibrillary tangles (NFTs). Several risk factors are identified or under investigation, including both genetic and environmental factors, as potential triggers of AD. AD may be classified as familial AD (FAD, at < 65 years of age) or sporadic AD (SAD, at > 65 years of age). Most of the knowledge of AD pathogenesis has been defined by studying FAD mutations. Some of the genetic targets are precursor protein amyloid-ß (*APP*) and presenilins (*PSEN1* and *PSEN2*) associated with increased FAD risk. The more common form of AD occurs sporadically (representing >90% of cases) [66]. Multi-faceted pathogenesis of SAD is associated with several risk factors such as old age and the presence of the apolipoprotein (*APOE*) gene ε4 allele and/or the recently identified genetic risk factor sortilin-related receptor (*SORL1)*. SORL1 is an APOE receptor with primary expression in neurons of the brain [67]. 

Zebrafish have human orthologous genes that play key roles in AD. The zebrafish genes *psen1* [42] and *psen2* [43] are human *PSEN1* and *PSEN2* orthologs, respectively, whereas the genes *appa* and *appb* are human *APP* co-orthologs [41]. The zebrafish genome also contains orthologous genes for gamma-secretase’s complex components, *PSENEN (psenen)* [47], *NCTN (ncstn)* [48], and *APH1b (aph1b)*. In addition, β-secretase orthologs (*BACE1* and *BACE2*) were also identified (*bace1* [44] and *bace2* [45]) in zebrafish. The zebrafish genome contains co-orthologs of the microtubule-associated tau protein (*MAPT*) gene, which encodes tau protein (*mapta*, and *maptb*) [68]. The human *APOE* and *SORL1* co-orthologs *apoea* and *apoeb* are also present in the zebrafish genome [37,50] and *sorl1* [51], respectively. 

### 3.2. Zebrafish and Parkinson’s Disease

Dopaminergic neuron degeneration, as well as the presence of Lewy bodies called intracytoplasmic inclusions, are neuropathological lesions associated with Parkinson’s disease (PD). Six genes associated with Parkinsonism have been identified, including *Parkin*, *DJ-1*, *PINK1*, *α-Synuclein*, *UCHL-1*, and *LRRK2* [69]. Although predominantly a motion disorder, PD is a mixed group of neurological conditions that are not capable of producing or controlling movement and cognitive impairment [70]. The human *PARK2* ortholog in zebrafish (*park2*) resides on chromosome 13, and encodes a protein of 458 amino acids (465 in humans) [53]. The *PINK1* zebrafish ortholog has 54% similarity, and an initial study reported a severe developmental phenotype in pink1 k/d zebrafish [54]. The *PARK7* zebrafish ortholog encodes a protein of 189 amino acids with 83% human DJ-1 identity [52].

### 3.3. Zebrafish and Huntington’s and Other Polyglutamine Diseases

Huntington’s disease (HD) is a monogenic neurodegenerative disease that follows an autosomal dominant pattern of huntingtin gene mutant form (*HTT*) inheritance. The mutation encodes for an abnormal trinucleotide that leads to glutamine (CAG) expansion at the HTT protein amino terminal and arises in an extended polyglutamine tract of the Huntingtin protein [71]. This causes cell death by gain of function mechanisms, like protein accumulation, mitochondrial dysfunction, and caspase activation. A decline in normal Huntingtin can also make a significant contribution to pathogenesis [72]. To try to elucidate the loss as well as the gain of function mechanisms, zebrafish models are being used. The HD cDNA homology in zebrafish was isolated as the first step towards discovering the possible role of the HD gene in initial vertebrate development [56]. This cDNA codes a predicted protein product of 3121 amino acids with a human HTT identity of 70%. Loss of developmental expression of 15/hd1 caused noticeable morphological abnormalities, including pericardial edema microcephalus and CNS necrosis [73]. Zebrafish *htt* is also necessary for normal pharyngeal arch cartilage development [74]. 

### 3.4. Zebrafish and Amyotrophic Lateral Sclerosis (ALS) 

Amyotrophic lateral sclerosis (ALS) is characterized by protein inclusions present in the affected neurons. These protein inclusions are linked to spinal cord motor neuron loss and downward motor tracts in the brain and spinal cord. Familial ALS is fairly rare, but a gene-encoding mutation of superoxide dismutase (*SOD1*) inherited in an autosomal dominant motif causes 20% of such cases [75,76]. The mutations are usually prescribed by gain of function mechanisms [77]. Over 150 mutations have been discovered in *SOD1*, including the point mutations G93R and G85R. Recent studies also indicate a role for *SOD1* in the sporadic form of ALS and propose a prion-like function of protein misfolding. Moreover, a few of the recently identified genes involved in ALS, such as *FUS* and *TARDBP*, also demonstrate a high tendency to act similar to prions in misfolding proteins.

A recent study used zebrafish to assess overexpression of *SOD1* by mRNA microinjection to study ALS etiology. In this study, vascular endothelial growth factor (*VEGF*) overexpression rescued the *SOD1*-expressing zebrafish axonopathy, while *VEGF* morpholino knockdown exacerbated the abnormalities [27]. However, one of the limitations in working with ALS in vivo models is the lack of comprehensive methods to assess the presymptomatic course of the disease. The zebrafish provides advantages in the study of processes of early disease with rapid development and reach post-embryonic life about 3 days post fertilization (dpf), which is similar to neonatal mouse development.

### 3.5. Zebrafish and Schizophrenia

Schizophrenia is a severe neurodegenerative disorder with the etiology of hallucination, delusions, depression, agitated body movements, confused thoughts and snafu speech, anhedonia, lack of motivation, and speech problems. The defects of schizophrenia are caused by early development in the brain [78]. About 1% of the world’s population is affected by schizophrenia and is characterized by neuronal dysfunction, which results in deficiencies in various cognitive areas including visual and verbal memory, learning, and attention [79]. Patients with schizophrenia, as well as with HD, have impaired preimpulse inhibition (PPI) [80,81], a type of sensorimotor gaiting [82]. PPI is a neurological event where the response following shocking stimulus is defeated by a weak prestimulus or prepulse and is conserved among vertebrates. The sensorimotor zebrafish gating has been described in 6 dpf larvae for PPI testing [83]. Twin studies have a projected heritage of around 81% for schizophrenia and an environmental impact of about 11% (factors such as diet, parenting, and exposure to toxins or teratogens) [84]. A large number of cases of schizophrenia are sporadic and appear in a family without a history of the disease [85]. Many genes have been linked to schizophrenia susceptibility. Genes with a largely robust disease connection include dystrobrevin binding protein 1 (*DTNBP1*), neuregulin1, disrupted in schizophrenia1 (*DISC1*), kinesin family member 1 (*KIF1*), kinesin family member 17 (*KIF17*), *SH3*, multiple ankyrin repeat domains 3 (*SHANK3*), and *NOTCH4* [86,87]. Candidate genes for schizophrenia may be vital in determining neuronal migration, neurogenesis, and cell fate [88]. 

Burgess and Granato [89] developed an endophenotype of schizophrenia in zebrafish PPI. Exposure to apomorphine and ketamine influences zebrafish PPI, and therefore appears to be facilitated by similar neurotransmitters as in other animals. The same study also identified five novel mutant lines with abnormal PPI responses. One of the most intensively studied genes associated with schizophrenia is *DISC1*, and was first identified with a high incidence of depression, schizophrenia, and bipolar disorder in a Scottish pedigree [90]. Furthermore, *disc1* studies in zebrafish have provided new information on this gene’s function.

### 3.6. Zebrafish and Epilepsy

Epilepsy is a common neurological disease caused by unexpected seizures that can vary from a short attention interval to severe and prolonged seizures and muscle cramps [91]. Epilepsy has a pathological mechanism that is poorly understood and is a complex brain disorder with many fundamental causes [92]. Zebrafish have a multifaceted nervous system with elegant behaviors, and are prone to seizure. Adult zebrafish have a wide array of established behaviors that can be studied, making them especially suitable for model development. The pentylenetetrazole (PTZ)-induced zebrafish epileptic seizure has been used to study the mechanism of epilepsy. The affordability of both larval and adult zebrafish, which allows the ontogenesis to investigate a wider range of epilepsy-related phenomena, is also useful. 

Several genetically altered zebrafish are now being used to study the behavior and brain function associated with epilepsy. Zebrafish (∼5–7 dpf) are commonly placed in multiple wells and tracked using video tracking software, continuously recorded by a camera. Mutations in two family members, Potassium Voltage-Gated Channel Subfamily Q Member 2 (*KCNQ2*) and Potassium Voltage-Gated Channel Subfamily Q Member 3 (*KCNQ3*), have been correlated with inherited neonatal epilepsy, e.g., benign family neonatal convulsions. These genes are highly expressed in zebrafish, providing support for studies of epilepsy using this vertebrate model [93]. 

## 4. Zebrafish Bioassay-Guided Isolation of Natural Product Drug Discovery

**Figure 1:**
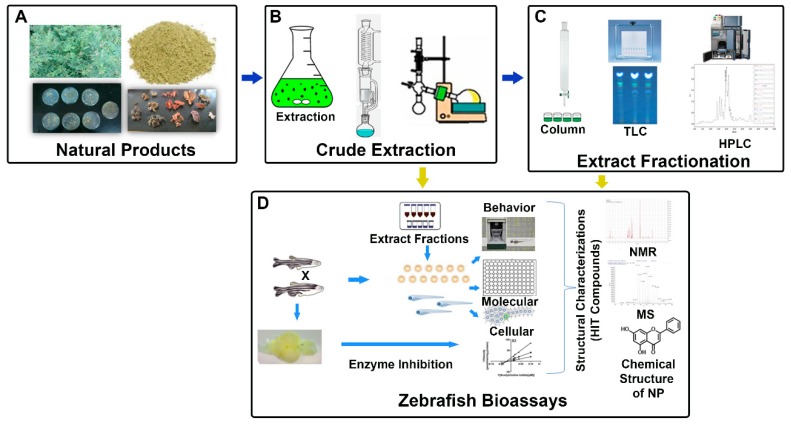
Schematic representation of zebrafish bioassay-guided isolation of natural products. (**A**) Different sources of natural products. (**B**) Crude extraction of natural products. (**C**) Purification of biologically active compounds from various chromatographic methods. (**D**) Various zebrafish biological assays and structural characterization of HIT compounds using different spectroscopic techniques. (HPLC: high performance liquid chromatography; MS: mass spectrometry; NMR: nuclear magnetic resonance spectrometry; NPs: natural products; and TLC: thin-layer chromatography).

Zebrafish was first suggested by Jones and Huffmann of the Oklahoma Research Foundation as a model for in vivo drug development in 1957, and soon thereafter zebrafish were first used to examine NP bioactivities. Zebrafish bioassay-guided identification of NPs in a number of neurological disorders can be an attractive approach for generating novel lead compounds (Figure 1). Over the past decade, zebrafish as a primary model for HTS in the scope of drug discovery for NPs for neurological disease has grown [16,94,95,96]. Zebrafish model profits combined with robust chromatographic and spectroscopic methods are creating a path to discover and further develop HIT compounds from various plant extracts [97,98].

Zebrafish has recently emerged as a strong model in a wide range of applications for rapid analysis of gene function and small molecular bioactivity [15]. Zebrafish are well-suited to identify therapeutically potential bioactive NPs (Table 2). Zebrafish were first proposed over fifty years ago as an in vivo model for the discovery of small molecules of drugs [99]. This preliminary study examined the utility of zebrafish embryos and larvae to screen both NPs and synthetic compounds. Zebrafish offers the opportunity of in vivo swift microgram-scale bioactivity evaluation of small molecules, an attractive feature combined with high-resolution fractionation technologies and analytical methods like UHPLC-TOF-MS and NMR microflow. A recent example is the bioassay-guided isolation of zebrafish with six spirostane glycosylated triterpene important for decoction and methanol extract anti-sizing activity from *Solanum torvum* aerial parts, which was discovered by Soura Challal [95] and his colleagues. In addition, the recently identified flavonoid-*trans*-tephrostachin inhibitory of acetylcholinesterase has also been isolated from the leaves of Indian herb *Tephrosia purpurea* by a zebrafish bioassay [96]. 

## 5. Development of Zebrafish Models for Neurological Disorders

In order to study the genes associated with various neurodegenerative disorders, the zebrafish has proven to be a perfect system where the genetic material is directly injected into the fertilized embryo. For instant study of gene function, effective techniques for the manipulation of gene expression are available [105]. By inserting genes into specific tissue promoters using vectors such as the Tol2 transposase system, transgenic zebrafish can be efficiently produced [106]. The Cre/loxP [107] and GAL4-UAS [108] gene function analysis systems can also be used at specific time points to generate conditionally expressed transgenics. The major disadvantage to induce specific mutations in the zebrafish genome was the unavailability of effective previous technologies. However, transcription activator-like effector nuclease (TALENS), zinc finger nucleases (ZFNs), and type II prokaryotic CRISPR (clustered regularly short palindromic repeats)/Cas systems for targeted gene sequences have been developed in recent times and are now being applied to create zebrafish transgenic models [109,110]. Furthermore, new technologies have expanded development of adult zebrafish and cell culture-based models.

### 5.1. Transgenic Zebrafish Models 

Due to the effortless screening of genes and small molecules in zebrafish, innovative genetic pathways that enable the development stages for isolating chemical modifiers can be obtained easily. [111,112,113,114]. More recently, it was suggested that many of these benefits could be applied to the study of human disease: high-content small molecular screens, genetic suppressor screens, in vivo disease progression observations, use of fluorescent reporters to identify interesting cell populations, and rapid hypothesis testing experiments in statistically robust larvae samples could provide valuable insight into disease pathogenesis or new therapeutic approaches [115,116]. In 2008, ZFNs were used to describe the first targeted gene knockout in zebrafish, and morpholinos were used to show gene knockdowns in neurodegenerative diseases (Table 3). For example, an *appb* knockdown study showed that the dramatic developmental defects in embryos and function of *appb* were needed for axonal outgrowth of motor neurons in zebrafish [117]. In addition, the *bace1* knockout zebrafish was generated by zinc finger nucleases. *bace1* mutants in the PNS are hypomyelinated, whereas the CNS is not affected [45]. Furthermore, study of the leucine-rich repeat kinase 2 (*LRRK2*) gene associated with PD is being studied in the zebrafish. Along with neuronal loss, the morpholino-mediated gene knockdown of *lrrk2* zebrafish also caused developmental disturbances in the eyes, lens, and otic vesicles, including axis curvature defects, eye abnormalities, and edema [118]. Since then, reverse genetic tools have seen unprecedented growth rates with the introduction of TALEN and CRISPR-Cas9 systems, including an *apoea* knockout for AD [119] and a *tardbp* knockin and *fus* knockin for ALS [40,120] (Table 3). Further development of transgenic models using the recently developed CRISPR technique is set to unravel a greater potential for zebrafish in gene knockdown and knockin studies.

### 5.2. Generation of a Neurodegenerative Model Using Amyloid-β42 (Aβ42) in the Adult Zebrafish Brain

**Figure 2:**
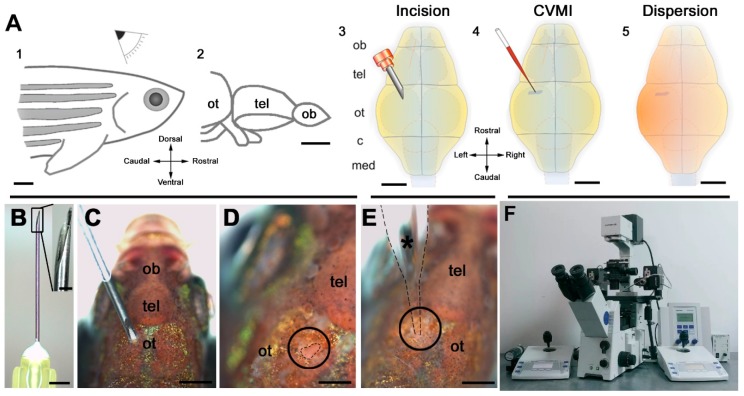
Outline of the pattern and its target regions of cerebroventricular microinjection (CVMI). (**A**) CVMI is achieved at the dorsal surface of the head (1) and targets, in this example, the forebrain that is rostral to the optic tectum (2). For injection, an incision is made into the skull over the optic tectum using a barbed-end canula (3). Through this slit, liquid is injected using a glass capillary (4). Injected liquid disperses rostrally (5). (**B**) The canula used for incision. (**C**) The incision on an adult fish (dorsal view). (**D**) The incision site marked by dashed lines. (**E**) Injection with the glass capillary (*) (dotted lines mark the outline). (**F**) Injection apparatus. Images (**A**–**E**) are adapted from [132]. (c: cerebellum; med: medulla; ob: olfactory bulb; ot: optic tectum; and tel: telencephalon)

The zebrafish bear extensive regenerative ability [126], and clinically important studies are aimed at understanding the mechanisms of zebrafish regeneration. Zebrafish are excellent tools because of their CNS regenerative capacity [127,128]. Models of neurodegeneration in the adult zebrafish brain will be helpful to investigate the activation state of the neural stem/progenitor cells (NSPCs) and to identify the molecular differences between zebrafish and mammalian NSPCs to utilize them for regenerative therapies [129]. Multifaceted inflammatory conditions in neurodegenerative diseases affect microglia, neurons, and NSPCs pleiotropically [130,131]. Kizil et al. first developed a gene knockdown method based on cerebroventricular microinjection (CVMI) in vivo morpholino oligonucleotide [132] in the adult zebrafish brain (Figure 2). CVMI injection in a skull incision is capable of uniformly targeting cells near the injection site, in this case the forebrain ventricular region containing neurogenic progenitor cells. The amyloid-β42 (Aβ42) induced neurotoxicity in adult zebrafish brain using CVMI of Aβ42 derivatives [133]. One of the earliest findings in understanding the etiology of AD was the discovery of a 40-length peptide in AD brains now called Aβ, which constitute the primary component of AD-related amyloid deposits [134,135]. Aβ is produced from the amyloid precursor protein (APP) with the continuous breakdown of β- and γ-secretases [136]. Importantly, the creation of Aβ through APP’s proteolytic processing is heterogeneous, leading to variable Aβ lengths, especially at the peptide’s carboxy terminus. 40 and 42 long residues are the two main forms of Aβ produced under normal APP processing conditions (Aβ40 and Aβ42, respectively). The shorter variety of Aβ40 is the majority of the Aβ produced in a normal individual [136]. Approximately 5%–15% of the total Aβ pool is Aβ42, and it is possible to observe smaller amounts of other Aβs, both longer and shorter. Generally, the brain’s Aβ pool has 5%–15% of Aβ42, which causes reminiscent phenotypes of amyloid pathophysiology: apoptosis, microglial activation, synaptic degeneration deficiencies, and learning. Aβ42 also results in NSPC proliferation and increased neurogenesis [37]. This understanding can help to design regenerative therapy-based drug discovery for neurological disorders. 

### 5.3. Zebrafish Cell Culture-Based Neurodegenerative Disease Models

The developing zebrafish embryo is an excellent source for culturing cells, including neural cells [137,138,139,140,141,142,143]. The technique to culture primary motor neuron (MN) in zebrafish has been developed for studying neurological disorders. The motor neuronal zebrafish cell culture was initiated at 24 hpf when the axonal development and outgrowth of MN started, allowing the development of MN axons in vivo in the context of the normal endogenous signs of the model organism, while also providing availability for an in vitro system. The zebrafish’s primary culture techniques offer another approach to examine the neuronal population. There have been reports of primary neuron culture protocols ranging from blastula stage to 19 hpf [144,145,146], but these cultures are derived from MN axon pathfinding and neuromuscular development prior to normal course. Primary MN axons in zebrafish are present at 18 hpf out of the spinal cord, while the appearance of secondary MN axonal path findings range from 26 to 34 hpf [147,148]. The brain explant cultures [149] and primary cell culture of muscle fibers [150,151,152] are possible to develop from the later development stages of zebrafish embryogenesis. The advantages of primary zebrafish cell culture provide a new foundation to develop potential therapies for neurological disorders. 

**Figure 3:**
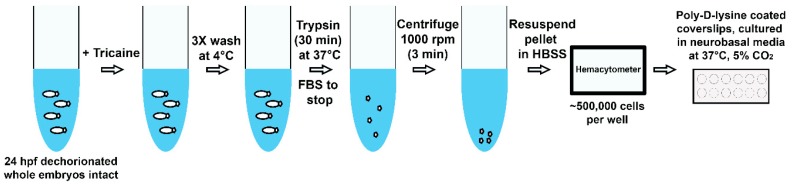
Summary of workflow for culturing zebrafish neurons. Embryos (24 hpf or 48 hpf) are collected, dechorionated, and placed in E3 medium and 16 μM tricaine microtubes. Embryos are then washed three times with an ice-cold E3 medium before being placed in 1 trypsin (in PBS) and pipeted for 30 minutes intermittently in a 37 °C water bath. To stop separation, fetal bovine serum (FBS) is then added and the tubes are centrifuged at 1000 rpm for 3 minutes. The supernatant is removed, and cell pellet resuspended in Hanks’ balanced salt solution (HBSS). Cells are counted using a hematocytometer, and ~500,000 cells are placed per well for culturing. It is recommended to change half of the media daily. (hpf: hours post fertilization)

A new protocol [153] outlines how the subcellular spreading and protein aggregation status of neurodegenerative disease-causing neurons from transgenic zebrafish embryos can be investigated (Figure 3). ALS and spinocerebellar ataxia type-3 (SCA3) can be studied from this protocol, as the disease-causing sarcoma-fused (FUS) and ataxin-3 proteins of the human variant gene can be expressed in the zebrafish cell culture. A combination of neuronal subtypes, including motor neurons, exhibited cultural differentiation as well as an outgrowth of neurites. The human mutant FUS mislocated from nuclei to cytosol, imitating observed in human ALS and the zebrafish FUS model. In contrast, zebrafish-grown neurons expressing human ataxin-3 with disease-associated improved polyQ repeats did not build up in nuclei as frequently reported in SCA3. Another simple and efficient protocol was recently proposed to obtain the primary cells of embryonic zebrafish [134]. By exploiting the cell-type rich resource specific fluorescent zebrafish reporter lines, different types of differentiated cells were cultivated and monitored, proving that they continued their original morphology in culture for many days and demonstrated that before cultivation, particular types of cells could be enriched with flow cytometry. This group also successfully tested several fluorescent vital colors to facilitate subcellular imaging. This technique delivers a new tool to enhance our understanding of neurodegenerative disorders pathogenesis and help the development of mechanism-based drugs for neurological disorders.

## 6. Conclusions

In summary, several observational studies have shown a connection between zebrafish and human neurological disorders. Zebrafish are proving to be an ideal model for screening pharmaceutical agents prior to testing in rodents. The long-term aims of this work are to clarify the mechanisms of neurodegeneration and develop new neuroprotective compounds for the treatment of neurodegenerative diseases. In adult zebrafish, the approach of neurodegeneration using Aβ peptides can also help to design regenerative therapies in the neurodegenerative situation. The described culture of neuronal cells adds a new tool to investigate neurodegenerative diseases regarding molecular and cellular mechanisms, high-quality live cell imaging, and the discovery of new therapeutic drugs for neurological disorders. The primary embryo of zebrafish and larvae culture has the potential to provide tremendous knowledge regarding various mechanisms and treatments for human disease. Zebrafish-based assays are capable of promoting the bioassay-guided fractionation of great numbers of bioactive extracts identified in these in vivo screens, thus allowing the isolation of different novel, bioactive natural products—most of which are likely to be desirable lead compounds for the development of new, potent drugs. These initial studies support zebrafish in helping to resolve a crucial bottleneck in the discovery of NPs by allowing rapid in vivo, microgram-scale, bioassay-guided fractionation analysis, and diverse natural extract dereplication studies.

## Figures and Tables

**Table 1:** Zebrafish orthologs of human genes involved in neurodegenerative disease pathogenesis.

Disease	Protein	Human Gene	Zebrafish Gene	Amino Acid Similarity (%)	Reference
Alzheimer’s Disease	Amyloid precursor protein	*APP* GeneID: 351 Locus: 21q21.2 Protein length: 695	*appa* GeneID: 58083 Chromosome: 1 Protein length: 738	74	[41]
			*appb* GeneID: 170846 Chromosome: 9 Protein length: 694	77	
	Presenilin-1	*PSEN1* GeneID: 5663 Locus: 14q24.3 Protein length: 467	*psen1* GeneID: 30221 Chromosome: 17 Protein length: 456	75	[42]
	Presenilin-2	*PSEN2* GeneID: 5664 Locus: 1q31-q42 Protein length: 448	*psen2* GeneID: 58026 Chromosome: 1 Protein length: 441	76	[43]
	β-secretase	*BACE1* GeneID: 23621 Locus: 11q23.2-q23.3 Protein length: 501	*bace1* zgc:77409 GeneID: 403005 Chromosome: 15 Protein length: 505	82	[44]
		*BACE2* GeneID: 25825 Locus: 21q22.2-q22.3 Protein length: 518	*bace2* zgc:103530 GeneID: 449818 chromosome: 15 Protein length: 462		[45]
	γ-secretase	*PSENEN* GeneID: 55851 Locus: 19q13.12 Protein length: 101	*psenen* GeneID: 402810 chromosome: 15 Protein length: 101	91	[46,47]
		*NCSTN* Gene ID: 23385 Locus: 1q23.2 Protein length: 709	*ncstn* GeneID: 494449 chromosome: 2 Protein length: 707	56	[48]
		*APH1b* Gene ID: 83464 Locus: 15q22.2 Protein length: 257	*aph1p* Gene ID: 386808 chromosome: 7 Protein length: 258		[46]
	Apolipoprotein E (ApoE)	*APOE* GeneID:348 Locus: 19q13.32 Protein length: 317	*apoea* Gene ID: 553587 chromosome: 19 Protein length: 269	27.5	[49]
			*apoeb* Gene ID: 30314 chromosome: 16 Protein length: 281		[50]
	Sortilin related receptor 1 (Sorl1)	*SORL1* GeneID: 6653, Locus:11q24.1 Protein length: 2214	*sorl1* Gene ID: 497306, chromosome: 15 Protein length: 2213	64	[51]
Parkinson’s Disease	DJ-1	*DJ-1* Gene ID: 11315 Locus: PARK7 1p36.23 Protein length: 189	*dj*-1 Gene ID: 449674 Chromosome: 11 Protein length: 189	83	[52]
	Parkin	*PRKN* Gene ID: 5071 Locus: PARK2 6q25.2-q27 Protein length: 465	*prkn* Gene ID: 550328 Chromosome: 13 Protein length: 458	62	[53]
	PTEN-induced kinase 1 (PINK-1)	*PINK1* Gene ID: 65018 Locus: PARK6 1p36 Protein length: 581	*pink1* Gene ID: 494085 Chromosome: 6 Protein length: 574	54	[54]
	Leucine-rich repeat kinase2 (LRRK2)	*LRRK2* GeneID: 120892 Locus: PARK8 12q12 Protein length: 2527	*lrrk2* GeneID: 559366 Chromosome: 25 Protein length: 1985	38	[55]
Huntington’s Disease	Huntingtin	*HTT* GeneID: 3064 Locus: 4q16.3 Protein length: 3144	*htt* GeneID: 30214 Chromosome: 1 Protein length: 3121	70	[56]
Amyotrophic Lateral Sclerosis (ALS)	Fused in sarcoma	*FUS* GeneID: 2521 Locus: 16p11.2 Protein length: 526	*fus* Gene ID: 394058 Chromosome: 3 Protein length: 541	63	[57]
	Tar DNA binding protein of 43 (TDP-43)	*TARDBP* GeneID: 23435 Locus: 1p36.22 Protein length: 414	*tardpb* GeneID: 325052 Chromosome: 6 Protein length: 412	71	[58]
Spinocerebellar Ataxia Type 1	Ataxin 1	*ATXN1* GeneID: 6310 Locus: 6p23 Protein length: 815	*atxn1a* GeneID: 565841 Chromosome: 16 Protein length: 827	32	[59]
			*atxn1b* GeneID: 557340 Chromosome: 19 Protein length: 781	42	
Schizophrenia	Dystrobrevin binding protein	*DISC1* Gene ID: 27185 Locus: 1q42.2 Protein length:854	*disc1* GeneID: 407621 Chromosome: 13 Protein length: 994	53	[60]
	Kinesin family member 17	*KIF17* Gene ID: 57576 Locus: 1p36.12 Protein length:1029	*kif17* GeneID:557863 Chromosome: 11 Protein length: 823	83	[61]

**Table 2:** Zebrafish bioassay-guided isolation and structurally characterized natural products.

Source	Disease/Targets	Molecules	References
*Pharbitis nil* (Seeds)	anti-seizure	Pharbitin	[100]
*Rehmannia glutinosa* (Root)	angiogenesis effect	Norviburtinal	[101]
*Rhynchosia viscosa* (Whole Plant)	angiogenesis effect	Rhynchoviscin	[98]
*Dysosma versipellis*	anti-angiogenesis	Kaempferol	[102]
*Ligusticum sinense* (Rhizoma)	anti-melanogenesis	1. Lignan 2. cis-4-pentylcyclohex-3-ene-1,2-diol	[103]
*Tephrosia purpurea* (Leaves)	anti-acetylcholinesterase	*trans*-Tephrostachin	[96]
*Solanum torvum* (leaves)	anti-convulsant	Paniculonin A Paniculonin B	[95]
*Skeletonema marinoi*	anti-seizure	Inosine	[104]

**Table 3:** Transgenic zebrafish models for neurodegenerative diseases.

Disease	Gene	Technique	References
**Alzheimer’s Disease**	*appb*	Morpholino injection - knockdown	[117]
	*psen1*	Morpholino injections - knockdown	[121]
	*bace2*	Zinc-finger nucleases (ZFNs)—knockout	[45]
	*psenen*	morpholino injection—knockdown	[47]
	*apoea*	CRISPR-Cas—knockout	[119]
	*apoe*	morpholino injection (live cell imaging)	[122]
**Parkinson’s Disease**	*djJ-1*	morpholino injection—knockdown	[123]
	*prkn*	gripNAsTM-mediated knockdown	[124]
	*pink1*	transcription activator-like effector nucleases (TALENs)—knockdown	[125]
	*lrrk2*	morpholino injection—knockdown	[118]
**Amyotrophic Lateral Sclerosis**	*tardbp (bpt1)*	CRISPR-Cas—knockin	[40]
	*fus*	CRISPR-Cas—knockin	[120]
**Huntington’s Disease**	*htt*	morpholino injection—knockdown	[71]

## References

[1] Devine M.J., Plun-Favreau H., Wood N.W. (2011). Parkinson’s disease and cancer: Two wars, one front. Nat. Rev. Cancer.

[2] Tabarés-seisdedos R., Rubenstein J.L. (2013). Inverse cancer comorbidity a serendipitous opportunity to gain insight into CNS disorders. Nat. Rev. Neurosci..

[3] Behrens M.I., Silva M., Salech F., Ponce D.P., Merino D., Sinning M., Xiong C., Roe M.C., Quest A.F.G. (2012). Inverse susceptibility to oxidative death of lymphocytes obtained from Alzheimer’s patients and skin cancer survivors: Increased apoptosis in Alzheimer’s and reduced necrosis in cancer. J. Gerontol. Ser. A Biol Sci. Med. Sci..

[4] Hilario E., Álvarez A., Domínguez A., Suarez-Merino B., Goñi-de-Cerio F. (2013). Central nervous system diseases and the role of the blood-brain barrier in their treatment. Neurosci. Discov..

[5] Gilmore J.L., Yi X., Quan L., Kabanov A.V. (2008). Novel Nanomaterials for Clinical Neuroscience. J. Neuroimmune Pharmacol..

[6] Rosén J., Gottfries J., Muresan S., Backlund A., Oprea T.I. (2009). Novel Chemical Space Exploration via Natural Products. J. Med. Chem..

[7] Koehn F.E., Carter G.T. (2005). The evolving role of natural products in drug discovery. Nat. Rev. Drug Discov..

[8] Harvey A.L. (2008). Natural products in drug discovery. Drug Discov. Today.

[9] Newman D.J., Cragg G.M. (2012). Natural Products As Sources of New Drugs over the 30 Years from 1981 to 2010. J. Nat. Prod..

[10] Macarron R., Banks M.N., Bojanic D., Burns D.J., Cirovic D.A., Garyantes T., Green D.V.S., Hertzberg R.P., Janzen W.P., Paslay J.W. (2011). Impact of high-throughput screening in biomedical research. Nat. Rev. Drug Discov..

[11] Welsch M.E., Snyder S.A., Stockwell B.R. (2010). Privileged scaffolds for library design and drug discovery. Curr. Opin. Chem. Biol..

[12] Cordell G.A. (2000). Biodiversity and drug discovery—A symbiotic relationship. Phytochemistry.

[13] Vuorela P., Tammela P., Rauha J., Saikku P., Leinonen M., Vuorela H. (2004). Natural Products in the Process of Finding New Drug Candidates. Curr. Med. Chem..

[14] Testa R., Bonfigli A., Genovese S., De Nigris V., Ceriello A. (2016). The Possible Role of Flavonoids in the Prevention of Diabetic Complications. Nutrients.

[15] Zon L.I., Peterson R.T. (2005). In vivo drug discovery in the zebrafish. Nat. Rev. Drug Discov..

[16] Crawford A.D., Esguerra C.V., de Witte P.A.M. (2008). Fishing for Drugs from Nature: Zebrafish as a Technology Platform for Natural Product Discovery. Planta Med..

[17] Hu J.-F., Yoo H.-D., Williams C.T., Garo E., Cremin P.A., Zeng L., Vervoort H.C., Lee C.M., Hart S.M., Goering M.G. (2005). Miniaturization of the Structure Elucidation of Novel Natural Products—Two Trace Antibacterial Acylated Caprylic Alcohol Glycosides from Arctostaphylos pumila. Planta Med..

[18] Choi D.-K., Lee K., More S.V., Kumar H., Kang S.M., Song S.-Y. (2013). Advances in Neuroprotective Ingredients of Medicinal Herbs by Using Cellular and Animal Models of Parkinson’s Disease. Evid. Based Complement. Altern Med..

[19] Zhang Z.J., Cheang L.C.V., Wang M.W., Lee S.M.Y. (2011). Quercetin exerts a neuroprotective effect through inhibition of the iNOS/NO system and pro-inflammation gene expression in PC12 cells and in zebrafish. Int. J. Mol. Med..

[20] Chan H.M., Guo B.J., Sa F., Li S., Lee S.M.Y., Zhang Z.J., Zheng Y. (2015). Pharmacokinetic Study and Optimal Formulation of New Anti-Parkinson Natural Compound Schisantherin, A. Parkinsons Dis..

[21] Löscher W., Potschka H. (2005). Role of drug efflux transporters in the brain for drug disposition and treatment of brain diseases. Prog. Neurobiol..

[22] Jeffrey P., Summerfield S. (2010). Assessment of the blood–brain barrier in CNS drug discovery. Neurobiol. Dis..

[23] Taylor J.P., Hardy J., Fischbeck K.H. (2002). Toxic proteins in neurodegenerative disease. Science.

[24] Bandmann O., Burton E.A. (2010). Genetic zebrafish models of neurodegenerative diseases. Neurobiol. Dis..

[25] Babin P.J., Goizet C., Raldúa D. (2014). Zebrafish models of human motor neuron diseases: Advantages and limitations. Prog. Neurobiol..

[26] Lemay N., Hayward L.J., Bosco D.A., Brown R.H., Zhou H., Burke C., Kwiatkowski T.J., Sapp P., Yasek D.M., Brown R.H., Hayward L.J. (2010). Mutant FUS proteins that cause amyotrophic lateral sclerosis incorporate into stress granules. Hum. Mol. Genet..

[27] Lemmens R., Van Hoecke A., Hersmus N., Geelen V., D’Hollander I., Thijs V., Van Den Bosch L., Carmeliet P., Robberecht W. (2007). Overexpression of mutant superoxide dismutase 1 causes a motor axonopathy in the zebrafish. Hum. Mol. Genet..

[28] Laird A.S., Mackovski N., Rinkwitz S., Becker T.S., Giacomotto J. (2016). Tissue-specific models of spinal muscular atrophy confirm a critical role of SMN in motor neurons from embryonic to adult stages. Hum. Mol. Genet..

[29] Paquet D., Bhat R., Sydow A., Mandelkow E.-M., Berg S., Hellberg S., Fälting J., Schmid B., Haass C. (2009). A zebrafish model of tauopathy allows in vivo imaging of neuronal cell death and drug evaluation Find the latest version: Technical advance A zebrafish model of tauopathy allows in vivo imaging of neuronal cell death and drug evaluation. J. Clin. Investig..

[30] Miller V.M., Nelson R.F., Gouvion C.M., Williams A., Rodriguez-Lebron E., Harper S.Q., Davidson B.L., Rebagliati M.R., Paulson H.L. (2005). CHIP Suppresses Polyglutamine Aggregation and Toxicity In Vitro and In Vivo. J. Neurosci..

[31] McGown A., McDearmid J.R., Panagiotaki N., Tong H., Al Mashhadi S., Redhead N., Lyon A.N., Beattie C.E., Shaw P.J., Ramesh T.M. (2013). Early interneuron dysfunction in ALS: Insights from a mutant sod1 zebrafish model. Ann. Neurol..

[32] Kabashi E., Lin L., Tradewell M.L., Dion P.A., Bercier V., Bourgouin P., Rochefort D., Hadj S.B., Durham H.D., Rouleau C.V.V.G.A. (2009). Gain and loss of function of ALS-related mutations of TARDBP (TDP-43) cause motor deficits in vivo. Hum. Mol. Genet..

[33] Bai Q., Burton E.A. (2011). Zebrafish models of Tauopathy HHS Public Access. Biochim. Biophys. Acta.

[34] Hartl F.U., Kretzschmar H., Haass C., Hirschberger T., Giese A., Schmid B., Schiffer N.W., Broadley S.A., Tavan P. (2006). Identification of Anti-prion Compounds as Efficient Inhibitors of Polyglutamine Protein Aggregation in a Zebrafish Model. J. Biol. Chem..

[35] Charles B.K. (2002). Patterning the Brain of the Zebrafish Embryo. Annu. Rev. Neurosci..

[36] Wullimann M.F., Rupp B., Reichert H., Verlag B. (1996). Neuroanatomy of the Zebrafish Brain: A Topological Atlas.

[37] Brown J., Guerra-Assunção J.A., Saunders D., Willey D., Barker D., Ellwood M., Gordon D., Chow W., Clark R., Karotki L. (2013). The zebrafish reference genome sequence and its relationship to the human genome. Nature.

[38] Kumar S., Hedges B.S. (1998). Amolecular timescalefor vertebrateevolution Sudhir. Nature.

[39] Clarke A.R. (2003). Transgenesis Techniques: Principles and Protocols.

[40] Hruscha A., Krawitz P., Rechenberg A., Heinrich V., Hecht J., Haass C., Schmid B. (2013). Efficient CRISPR/Cas9 genome editing with low off-target effects in zebrafish. Development.

[41] Musa A., Lehrach H., Russo V.E. (2001). Distinct expression patterns of two zebrafish homologues of the human APP gene during embryonic development. Dev. Genes Evol..

[42] Leimer U., Lun K., Romig H., Walter J., Grünberg J., Brand M., Haass C. (1999). Zebrafish (Danio rerio) Presenilin Promotes Aberrant Amyloid β-Peptide Production and Requires a Critical Aspartate Residue for Its Function in Amyloidogenesis. Biochemistry.

[43] Groth C., Nornes S., McCarty R., Tamme R., Lardelli M. (2002). Identification of a second presenilin gene in zebrafish with similarity to the human Alzheimer’s disease gene presenilin2. Dev. Genes Evol..

[44] Moussavi Nik S.H., Wilson L., Newman M., Croft K., Mori T.A., Musgrave I., Lardelli M. (2012). The BACE1-PSEN-AβPP regulatory axis has an ancient role in response to low oxygen/oxidative stress. J. Alzheimer’s Dis..

[45] Van Bebber F., Hruscha A., Willem M., Schmid B., Haass C. (2013). Loss of Bace2 in zebrafish affects melanocyte migration and is distinct from Bace1 knock out phenotypes. J. Neurochem..

[46] Francis R., McGrath G., Zhang J., Ruddy D.A., Sym M., Apfeld J., Nicoll M., Maxwell M., Hai B., Ellis M.C. (2002). aph-1 and pen-2 are required for Notch pathway signaling, γ-secretase cleavage of βAPP, and presenilin protein accumulation. Dev. Cell.

[47] Campbell W.A., Yang H., Zetterberg H., Baulac S., Sears J.A., Liu T., Wong S.T.C., Zhong T.P., Xia W. (2006). Zebrafish lacking Alzheimer presenilin enhancer 2 (Pen-2) demonstrate excessive p53-dependent apoptosis and neuronal loss. J. Neurochem..

[48] Team MGC (MGC) P. (2002). Generation and initial analysis of more than 15,000 full-length human and mouse cDNA sequences. Proc. Natl. Acad. Sci. USA.

[49] Babin P.J., Thisse C., Durliat M., Andre M., Akimenko M.-A., Thisse B. (1997). Both apolipoprotein E and A-I genes are present in a nonmammalian vertebrate and are highly expressed during embryonic development. Proc. Natl. Acad. Sci. USA.

[50] Woods I.G., Wilson C., Friedlander B., Chang P., Reyes D.K., Nix R., Kelly P.D., Chu F., Postlethwait J.H., Talbot W.S. (2005). The zebrafish gene map defines ancestral vertebrate chromosomes. Genome Res..

[51] Lee J., Peterson S.M., Freeman J.L. (2017). Sex-specific characterization and evaluation of the Alzheimer’s disease genetic risk factor sorl1 in zebrafish during aging and in the adult brain following a 100 ppb embryonic lead exposure. J. Appl. Toxicol..

[52] Bai Q., Mullett S.J., Garver J.A., Hinkle D.A., Burton E.A. (2006). Zebrafish DJ-1 is evolutionarily conserved and expressed in dopaminergic neurons. Brain Res..

[53] Flinn L., Mortiboys H., Volkmann K., Kster R.W., Ingham P.W., Bandmann O. (2009). Complex i deficiency and dopaminergic neuronal cell loss in parkin-deficient zebrafish (Danio rerio). Brain.

[54] Anichtchik O., Roach A., Goldsmith P., Diekmann H., Rubinsztein D.C., Fleming A. (2008). Loss of PINK1 Function Affects Development and Results in Neurodegeneration in Zebrafish. J. Neurosci..

[55] Ren G., Xin S., Li S., Zhong H., Lin S. (2011). Disruption of lrrk2 does not cause specific loss of dopaminergic neurons in zebrafish. PLoS ONE.

[56] Karlovich C.A., John R.M., Ramirez L., Stainier D.Y., Myers R.M. (1998). Characterization of the Huntington’s disease (HD) gene homolog in the zebrafish Danio rerio. Gene.

[57] Laboissonniere L.A., Smith C.L., Mesenbrink J., Chowdhury R., Burney A., Lang M., Sierra M., Stark A., Maldonado-Casalduc G., Muller M. (2018). ALS-associated genes display CNS expression in the developing zebrafish. Gene Expr. Patterns.

[58] Schmid B., Hruscha A., Hogl S., Banzhaf-Strathmann J., Strecker K., van der Zee J., Teucke M., Eimer S., Hegermann J., Kittelmann M. (2013). Loss of ALS-associated TDP-43 in zebrafish causes muscle degeneration, vascular dysfunction, and reduced motor neuron axon outgrowth. Proc. Natl. Acad. Sci. USA.

[59] Carlson K.M., Melcher L., Lai S., Zoghbi H.Y., Clark H.B., Orr H.T. (2009). Characterization of the zebrafish atxn1/axh gene family. J. Neurogenet..

[60] Wood J.D., Bonath F., Kumar S., Ross C.A., Cunliffe V.T. (2009). Disrupted-in-schizophrenia 1 and neuregulin 1 are required for the specification of oligodendrocytes and neurones in the zebrafish brain. Hum. Mol. Genet..

[61] Kabashi E., Brustein E., Champagne N., Drapeau P. (2011). Zebrafish models for the functional genomics of neurogenetic disorders. Biochim. Biophys. Acta.

[62] (2018). The State of the Art of Dementia Research: New Frontiers.

[63] Voisin T., Vellas B. (2009). Diagnosis and Treatment of Patients with Severe Alzheimer’s Disease. Drugs Aging.

[64] Regeur L., Badsberg Jensen G., Pakkenberg H., Evans S.M., Pakkenberg B. (1994). No global neocortical nerve cell loss in brains from patients with senile dementia of Alzheimer’s type. Neurobiol. Aging.

[65] Mark J., Paul D., Dorothy G., Juan C. (1994). Differences in the pattern of hippocampal neuronal loss in normal ageing and Alzheimer â€^TM^ s disease. Lancet.

[66] Blennow K., De Leon M.J., Zetterberg H. (2006). SeminarAlzheimer’s disease. Lancet.

[67] Jacobsen L., Madsen P., Jacobsen C., Nielsen M.S., Gliemann J., Petersen C.M. (2001). Activation and Functional Characterization of the Mosaic Receptor SorLA/LR11. J. Biol. Chem..

[68] Chen M., Martins R.N., Lardelli M. (2009). Complex splicing and neural expression of duplicated tau genes in zebrafish embryos. J. Alzheimer’s Dis..

[69] Abeliovich A., Flint Beal M. (2006). Parkinsonism genes: Culprits and clues. J. Neurochem..

[70] Galvin J.E. (2006). Cognitive change in Parkinson disease. Alzheimer Dis. Assoc. Disord..

[71] Huntington T., Macdonald M.E., Ambrose C.M., Duyao M.P., Myers R.H., Lin C., Srinidhi L., Barnes G., Taylor S.A., James M. (1993). A Novel Gene Containing a Trinucleotide Repeat That Is Expanded and Unstable on Huntington’s Disease Chromosomes. Cell.

[72] Walker F.O. (2007). Huntington’s disease. Lancet.

[73] Lumsden A.L., Henshall T.L., Dayan S., Lardelli M.T., Richards R.I. (2007). Huntingtin-deficient zebrafish exhibit defects in iron utilization and development. Hum. Mol. Genet..

[74] Diekmann H., Fleming A., Rubinsztein D.C., Roach A., Anichtchik O., Goldsmith P., Futter M. (2009). Decreased BDNF Levels Are a Major Contributor to the Embryonic Phenotype of Huntingtin Knockdown Zebrafish. J. Neurosci..

[75] Deng H.X., Hentati A., Tainer J.A., Iqbal Z., Cayabyab A., Hung W.Y., Getzoff E.D., Hu P., Herzfeldt B., Roos R.P. (1993). Amyotrophic lateral sclerosis and structural defects in Cu,Zn superoxide dismutase. Science.

[76] Rosen D.R., Siddiquet T., Pattersont D., Figlewicz D.A., Ii P.S., Hentatit A., Donaldsont D., Goto J., Ii J.P.O.R., Dengt H. (1993). Mutations in Cu/Zn superoxide dismutase gene are associated with familial amyotrophic lateral sclerosis. Nature.

[77] Turner B.J., Talbot K. (2008). Transgenics, toxicity and therapeutics in rodent models of mutant SOD1-mediated familial ALS. Prog. Neurobiol..

[78] Weinberger D. (1995). From neuropathology to neurodevelopment. Lancet.

[79] Nuechterlein K.H., Barch M., Gold J.M., Goldberg T.E., Green M.F., Heaton R.K. (2004). Identification of separable cognitive factors in schizophrenia. Schizophr. Res..

[80] Swerdlow N.R., Butters N., Geyer M.A., Paulsen J., Swenson M.R., Braff D.L. (2008). Impaired prepulse inhibition of acoustic and tactile startle response in patients with Huntington’s disease. J. Neurol. Neurosurg. Psychiatry.

[81] Braff D.L., Geyer M.A., Swerdlow N.R. (2001). Human studies of prepulse inhibition of startle: Normal subjects, patient groups, and pharmacological studies. Psychopharmacology.

[82] Braff D., Schork N.J. (2007). Gottesman, I.I. Endophenotyping schizophrenia. Am. J. Psychiatry.

[83] Burgess H.A., Granato M. (2007). Sensorimotor Gating in Larval Zebrafish. J. Neurosci..

[84] Sullivan P.F., Kendler K.S., Neale M.C. (2003). Schizophrenia as a Complex Trait. Arch. Gen. Psychiatry.

[85] Van Rensburg E.J., Xu B., Karayiorgou M., Levy S., Roos J.L., Gogos J.A. (2008). Strong association of de novo copy number mutations with sporadic schizophrenia. Nat. Genet..

[86] Tarabeux J., Champagne N., Brustein E., Hamdan F.F., Gauthier J., Lapointe M., Maios C., Piton A., Spiegelman D., Henrion É.M., Millet B. (2010). De Novo Truncating Mutation in Kinesin 17 Associated with Schizophrenia. Biol. Psychiatry.

[87] Girard S.L., Dion P.A., Rouleau G.A. (2012). Schizophrenia genetics: Putting all the pieces together. Curr. Neurol. Neurosci. Rep..

[88] Morris J.A. (2009). Zebrafish: A model system to examine the neurodevelopmental basis of schizophrenia. Prog. Brain Res..

[89] Burgess H.A., Granato M. (2007). Modulation of locomotor activity in larval zebrafish during light adaptation. J. Exp. Biol..

[90] Millar J.K., Christie S., Semple C.A.M., Porteous D.J. (2000). Chromosomal Location and Genomic Structure of the Human Translin-Associated Factor X Gene (TRAX; TSNAX) Revealed by Intergenic Splicing to DISC1, a Gene Disrupted by a Translocation Segregating with Schizophrenia. Genomics.

[91] Zashikhina A. (2014). Juvenile Chronic Physical Illness in Northern Russia: Studies on Mental Health, Health-Related Quality of Life, and Family Functioning. Ph.D. Thesis.

[92] Galanopoulou A.S., Buckmaster P.S., Staley K.J., Moshe S.L., Perucca E., Engel J., Löscher W., Noebels J.L., Pitkänen A., Stables J., White H.S. (2012). Identification of new epilepsy treatments: Issues in preclinical methodology. Epilepsia.

[93] Desmond D., Kyzar E., Gaikwad S., Green J., Riehl R., Roth A., Stewart A.M., Kalueffet A.V. (2012). Assessing epilepsy-related behavioral phenotypes in adult zebrafish. Zebrafish Protocols for Neurobehavioral Research.

[94] Crawford A.D., Liekens S., Kamuhabwa A.R., Maes J., Munck S., Busson R., Rozenski J., Esguerra C.V., de Witte P.A.M. (2011). Zebrafish bioassay-guided natural product discovery: Isolation of angiogenesis inhibitors from East African medicinal plants. PLoS ONE.

[95] Marcourt L., Bock M., Maljevic S., Dayrit F.M., Challal S., Queiroz E.F., de Witte P.A.M., Crawford A.D., Harvey A.L., Buenafe O.E.M. (2014). Zebrafish Bioassay-Guided Microfractionation Identifies Anticonvulsant Steroid Glycosides from the Philippine Medicinal Plant Solanum torvum. ACS Chem. Neurosci..

[96] Pitchai A., Nagarajan N., Vincent S.G.P., Rajaretinam R.K. (2018). Zebrafish bio-assay guided isolation of human acetylcholinesterase inhibitory trans-tephrostachin from Tephrosia purpurea (L.) Pers. Neurosci. Lett..

[97] Buenafe O.E., Orellana-Paucar A., Maes J., Huang H., Ying X., De Borggraeve W., Crawford A.D., Luyten W., Esguerra C.V., De Witte P. (2013). Tanshinone IIA exhibits anticonvulsant activity in zebrafish and mouse seizure models. ACS Chem. Neurosci..

[98] Esguerra C.V., Kamuhabwa A.R., Wolfender J.-L., Moshi M.J., Siverio-Mota D., Marcourt L., Maes J., Bohni N., de Witte P.A.M., Cordero-Maldonado M.L. (2013). Integration of Microfractionation, qNMR and Zebrafish Screening for the In Vivo Bioassay-Guided Isolation and Quantitative Bioactivity Analysis of Natural Products. PLoS ONE.

[99] Jones R.W., Huffman M.N. (1957). Fish Embryos as Bio-Assay Material in Testing Chemicals for Effects on Cell Division and Differentiation. Trans. Am. Microsc. Soc..

[100] Liu M., Copmans D., Lu J.G., Yang M.R., Sourbron J., Ny A., Jiang Z.H., de Witte P.A.M., Luyten W. (2019). Bioassay-guided isolation of anti-seizure principles from Semen Pharbitidis using a zebrafish pentylenetetrazol seizure model. J. Ethnopharmacol..

[101] Liu C.L., Cheng L., Kwok H.F., Ko C.H., Lau T.W., Koon C.M., Zhao M., Lau C.P., Lau K.M., Wong C.W. (2011). Bioassay-guided isolation of norviburtinal from the root of Rehmannia glutinosa, exhibited angiogenesis effect in zebrafish embryo model. J. Ethnopharmacol..

[102] Liang F., Han Y., Gao H., Xin S., Chen S., Wang N., Qin W., Zhong H., Lin S., Yao X. (2015). Kaempferol Identified by Zebrafish Assay and Fine Fractionations Strategy from Dysosma versipellis Inhibits Angiogenesis through VEGF and FGF Pathways. Sci. Rep..

[103] Cheng M.-C., Lee T.-H., Chu Y.-T., Syu L.-L., Hsu S.-J., Cheng C.-H., Lee J., Wu C.-K. (2018). Melanogenesis Inhibitors from the Rhizoma of Ligusticum Sinense in B16-F10 Melanoma Cells In Vitro and Zebrafish In Vivo. Int. J. Mol. Sci..

[104] Brillatz T., Lauritano C., Jacmin M., Khamma S., Marcourt L., Righi D., Romano G., Esposito F., Ianora A., Queiroz E.F. (2018). Zebrafish-based identification of the antiseizure nucleoside inosine from the marine diatom Skeletonema marinoi. PLoS ONE.

[105] Thermes V., Grabher C., Ristoratore F., Bourrat F., Choulika A., Wittbrodt J., Joly J.-S., Thermes V., Grabher C., Ristoratore F. (2002). I-SceI meganuclease mediates highly efficient transgenesis in fish. Mech. Dev..

[106] Kawakami K., Shima A., Kawakami N. (2002). Identification of a functional transposase of the Tol2 element, an Ac-like element from the Japanese medaka fish, and its transposition in the zebrafish germ lineage. Proc. Natl. Acad. Sci. USA.

[107] Hans S., Kaslin J., Freudenreich D., Brand M. (2009). Temporally-controlled site-specific recombination in zebrafish. PLoS ONE.

[108] Halpern M.E., Rhee J., Goll M.G., Akitake C.M., Parsons M., Leach S.D. (2008). Gal4/UAS Transgenic Tools and Their Application to Zebrafish. Zebrafish.

[109] Schmid B., Haass C. (2013). Genomic editing opens new avenues for zebrafish as a model for neurodegeneration. J. Neurochem..

[110] Hwang W.Y., Fu Y., Reyon D., Maeder ML., Tsai S.Q., Sander J.D., Joung J.K., Peterson R.T., Yeh J.R.J. (2013). Efficient genome editing in zebrafish using a CRISPR-Cas system. Nat. Biotechnol..

[111] Burns C.G., Milan D.J., Grande E.J., Rottbauer W., MacRae C.A., Fishman M.C. (2005). High-throughput assay for small molecules that modulate zebrafish embryonic heart rate. Nat. Chem. Biol..

[112] Goldsmith P. (2004). Zebrafish as a pharmacological tool: The how, why and when. Curr. Opin. Pharmacol..

[113] Sun Z., Chen W., Farrington S., Haldi M., Hopkins N., Amsterdam A., Golling G., Townsend K., Burgess S. (2002). A large-scale insertional mutagenesis screen in zebrafish. Genes Dev..

[114] Guo S., Wilson S.W., Cooke S., Chitnis A.B., Driever W., Rosenthal A. (1999). Mutations in the Zebrafish Unmask Shared Regulatory Pathways Controlling the Development of Catecholaminergic Neurons. Dev. Biol..

[115] Amatruda J.F., Tallarico J.A., Weber G., Zon L.I., Pfaff K.L., King R.W., Straub C.T., Shepard J.L., Murphey R.D., Stern H.M. (2005). Small molecules that delay S phase suppress a zebrafish bmyb mutant. Nat. Chem. Biol..

[116] MacRae C.A., Milan D.J., Fishman M.C., Schreiber S.L., Peterson R.T., Peterson T.A., Shaw S.Y., Zhong T.P. (2004). Chemical suppression of a genetic mutation in a zebrafish model of aortic coarctation. Nat. Biotechnol..

[117] Song P., Pimplikar S.W. (2012). Knockdown of amyloid precursor protein in zebrafish causes defects in motor axon outgrowth. PLoS ONE.

[118] Prabhudesai S., Bensabeur F.Z., Abdullah R., Basak I., Baez S., Alves G., Holtzman N.G., Larsen J.P., Møller S.G. (2016). LRRK2 knockdown in zebrafish causes developmental defects, neuronal loss, and synuclein aggregation. J. Neurosci. Res..

[119] Wang W.Y., Fu Y., Reyon D., Maeder M.L., Kaini P., Sander J.D., Joung J.K., Peterson R.T., Yeh J.R.J. (2013). Heritable and Precise Zebrafish Genome Editing Using a CRISPR-Cas System. PLoS ONE.

[120] Armstrong G.A.B., Liao M., You Z., Lissouba A., Chen B.E., Drapeau P. (2016). Homology directed knockin of point mutations in the zebrafish tardbp and fus genes in ALS using the CRISPR/Cas9 system. PLoS ONE.

[121] Nery L.R., Silva N.E., Fonseca R., Vianna M.R.M. (2017). Presenilin-1 Targeted Morpholino Induces Cognitive Deficits, Increased Brain Aβ1−42 and Decreased Synaptic Marker PSD-95 in Zebrafish Larvae. Neurochem. Res..

[122] Peri F., Nüsslein-Volhard C. (2008). Live Imaging of Neuronal Degradation by Microglia Reveals a Role for v0-ATPase a1 in Phagosomal Fusion In Vivo. Cell.

[123] Bretaud S., Allen C., Ingham P.W., Bandmann O. (2007). p53-dependent neuronal cell death in a DJ-1-deficient zebrafish model of Parkinson’s disease. J. Neurochem..

[124] Fett M.E., Pilsl A., Paquet D., van Bebber F., Haass C., Tatzelt J., Schmid B., Winklhofer K.F. (2010). Parkin is protective against proteotoxic stress in a transgenic zebrafish model. PLoS ONE.

[125] Zhang Y., Nguyen D.T., Olzomer E.M., Poon G.P., Cole N.J., Puvanendran A., Phillips B.R., Hesselson D. (2017). Rescue of Pink1 Deficiency by Stress-Dependent Activation of Autophagy. Cell Chem. Biol..

[126] Tanaka E.M., Ferretti P. (2009). Considering the evolution of regeneration in the central nervous system. Nat. Rev. Neurosci..

[127] Chapouton P., Jagasia R., Bally-Cuif L. (2007). Adult neurogenesis in non-mammalian vertebrates. BioEssays.

[128] Kizil C., Kaslin J., Kroehne V., Brand M. (2012). Adult neurogenesis and brain regeneration in zebrafish. Dev. Neurobiol..

[129] Cosacak M.I., Papadimitriou C., Kizil C. (2015). Regeneration, Plasticity, and Induced Molecular Programs in Adult Zebrafish Brain. Biomed. Res. Int..

[130] Das S., Basu A. (2008). Inflammation: A new candidate in modulating adult neurogenesis. J. Neurosci. Res..

[131] Kizil C., Kyritsis N., Brand M. (2015). Effects of inflammation on stem cells: Together they strive?. EMBO Rep..

[132] Kizil C., Brand M. (2011). Cerebroventricular microinjection (CVMI) into adult zebrafish brain is an efficient misexpression method for forebrain ventricular cells. PLoS ONE.

[133] Bhattarai P., Thomas A.K., Cosacak M.I., Papadimitriou C., Mashkaryan V., Froc C., Reinhardt S., Kurth T., Dahl T., Zhang Y. (2016). IL4/STAT6 Signaling Activates Neural Stem Cell Proliferation and Neurogenesis upon Amyloid-β42 Aggregation in Adult Zebrafish Brain. Cell Rep..

[134] Wong C.W., Quaranta V., Glenner G.G. (1985). Neuritic plaques and cerebrovascular amyloid in Alzheimer disease are antigenically related. Proc. Natl. Acad. Sci. USA.

[135] Masters C.L., Simms G., Weinman N.A., Multhaup G., McDonald B.L., Beyreuther K. (1985). Amyloid plaque core protein in Alzheimer disease and Down syndrome. Proc. Natl. Acad. Sci. USA.

[136] Younkin S.G. (1998). The role of Aβ42 in Alzheimer’s disease. J. Physiol. Paris.

[137] Liu W., Guan Y., Collodi P. (2010). A Zebrafish Cell Culture Assay for the Identification of MicroRNA Targets. Zebrafish.

[138] Myhre J.L., Pilgrim D.B. (2010). Cellular Differentiation in Primary Cell Cultures from Single Zebrafish Embryos as a Model for the Study of Myogenesis. Zebrafish.

[139] Robles V., Martí M., Belmonte J.C.I. (2011). Study of Pluripotency Markers in Zebrafish Embryos and Transient Embryonic Stem Cell Cultures. Zebrafish.

[140] Sakowski S.A., Lunn J.S., Busta A.S., Palmer M., Dowling J.J., Feldman E.L. (2012). A novel approach to study motor neurons from zebrafish embryos and larvae in culture. J. Neurosci. Methods.

[141] Ciarlo C.A., Zon L. (2016). Embryonic cell culture in zebrafish. Methods Cell Biol..

[142] Sassen W.A., Lehne F., Russo G., Wargenau S., Dübel S., Köster R.W. (2017). Embryonic zebrafish primary cell culture for transfection and live cellular and subcellular imaging. Dev. Biol..

[143] Giros B., Fassier C., Nothias F., Lumsden A., Hazan J., Scholpp S., Hutt J.A., Houart C., Schneider-Maunoury S. (2010). Zebrafish atlastin controls motility and spinal motor axon architecture via inhibition of the BMP pathway. Nat. Neurosci..

[144] Liu D., Westerfield M. (2018). Clustering of muscle acetylcholine receptors requires motoneurons in live embryos, but not in cell culture. J. Neurosci..

[145] Ghosh C., Liu Y., Ma C., Collodi P. (1997). Cell cultures derived from early zebrafish embryos differentiate in vitro into neurons and astrocytes. Cytotechnology.

[146] Andersen S.S.L. (2001). Preparation of dissociated Zebrafish spinal neuron cultures. Methods Cell Sci..

[147] Beattie C.E. (2000). Control of motor axon guidance in the zebrafish embryo. Brain Res. Bull..

[148] Eisen J.S. (1991). Motoneuronal development in the embryonic zebrafish. Development.

[149] Hendricks M., Jesuthasan S. (2007). Electroporation-based methods for in vivo, whole mount and primary culture analysis of zebrafish brain development. Neural Dev..

[150] Saint-amant L., Sprague S.M., Hirata H., Li Q., Cui W.W., Zhou W., Poudou O., Hume R.I., Kuwada J.Y. (2007). The Zebrafish ennui Behavioral Mutation Disrupts Acetylcholine Receptor Localization and Motor Axon Stability. Dev. Neurobiol..

[151] Currie P.D., Ferguson C., Westerfield M., Key B., Nixon S.J., Méry P.-F., Parton R.G., Hancock J.F., Wegner J. (2005). Zebrafish as a model for caveolin-associated muscle disease; caveolin-3 is required for myofibril organization and muscle cell patterning. Hum. Mol. Genet..

[152] Vreede A.P., Dowling J.J., Low S.E., Feldman E.L., Bonnemann C.G., Gibbs E.M., Kuwada J.Y. (2009). Loss of Myotubularin Function Results in T-Tubule Disorganization in Zebrafish and Human Myotubular Myopathy. PLoS Genet..

[153] Don E.K., Watchon M., Yuan K.C., Fifita J.A., Blair I.P., Nicholson G.A., Laird A.S., Acosta J.R., Goldsbury C., Svahn A.J. (2018). Neuronal cell culture from transgenic zebrafish models of neurodegenerative disease. Biol. Open.

